# JC Virus PCR Detection Is Not Infallible: A Fulminant Case of Progressive Multifocal Leukoencephalopathy with False-Negative Cerebrospinal Fluid Studies despite Progressive Clinical Course and Radiological Findings

**DOI:** 10.1155/2015/643216

**Published:** 2015-03-12

**Authors:** Mohamed-Ali Babi, William Pendlebury, Steven Braff, Waqar Waheed

**Affiliations:** ^1^Department of Neurological Sciences, University of Vermont Medical Center, Burlington, VT 05401, USA; ^2^Department of Pathology, University of Vermont Medical Center, Burlington, VT 05401, USA; ^3^Department of Radiology, University of Vermont Medical Center, Burlington, VT 05401, USA

## Abstract

We describe a case with a false-negative PCR-based analysis for JC virus in cerebrospinal fluid (CSF) in a patient with clinical and radiological findings suggestive of progressive multifocal leukoencephalopathy (PML) who was on chronic immunosuppressive therapy for rheumatoid arthritis. Our patient developed rapidly progressive global decline with clinical and radiographic findings suggestive of PML, but JC virus PCR in CSF was negative. The patient passed away 3 months from the onset of her neurological symptoms. Autopsy confirmed the diagnosis of PML with presence of JC-polyoma virus by immunohistochemical staining. This case highlights the potential of false-negative JC virus PCR in CSF when radiographic and clinical features are suggestive of “possible PML.” We review the plausible causes of potential false-negative CSF results and suggest that when the clinical presentation is suspicious for PML repeat CSF analysis utilizing ultrasensitive PCR assay and subsequent brain biopsy should be considered if CSF remains negative. Additionally, appropriate exclusion of other neurologic conditions is essential.

## 1. Introduction

Progressive multifocal leukoencephalopathy (PML) is a severe demyelinating disorder of the CNS caused by infection of the JC-polyoma virus. PML has classically been described in immune-compromised individuals, such as acquired immune deficiency syndrome (AIDS), but it also occurs in people on chronic immunosuppressive therapy for solid organ transplants, in people with hematological malignancies receiving antineoplastic therapy, and in individuals with autoimmune diseases such as multiple sclerosis, rheumatoid arthritis, psoriasis, and systemic lupus erythematosus with or without immunosuppressive or immunomodulatory therapies that depress the immune response and allow JC virus reactivation [1–3]. However, PML has also rarely been described in individuals with minimal or no immunodeficiency identified such as hepatic cirrhosis, renal failure, and pregnancy suggesting that it is not only the immunosuppressive therapies but also the abnormal immune system which contributes to the vulnerability to JC virus infection or reactivation [1–3].

Some of the reported immunosuppressive therapies described in association with PML include efalizumab, natalizumab, infliximab, rituximab, belatacept, fludarabine, glucocorticoids, cytotoxic chemotherapy, and various transplant drugs such as tacrolimus and mycophenolate [4–10]. In some cases, combination immunosuppression therapies have also been implicated. However, and to the best of our knowledge, only one case report in the literature described association of adalimumab therapy with PML and none implicated concurrent adalimumab and methotrexate therapy with PML [6, 11].

Among patients with autoimmune diseases, but without HIV or malignancy, one study estimated PML incidence at 0.2/100,000 [2]. Another study reported the incidence rate of PML as 2.3 per 100,000 person-years in patients exposed to biological agents and 0.8 per 100,000 years in those not exposed to biological agents [2, 11].

The diagnosis of PML is suggested by clinical and neuroimaging features and further established by demonstrating the presence of JC virus DNA in the cerebrospinal fluid using polymerase chain reaction (PCR) [1, 3]. Although CSF PCR for JC virus is highly specific (92–99%) and sensitive (74–93%), false negatives do occur [12, 13]. Different treatment regimens have been suggested in the literature to address PML. However, the course of PML is usually progressive and fatal, albeit with variability of length of survival [9].

We describe a case of PML with false-negative CSF JC virus PCR, thus reinforcing the importance of obtaining histological confirmation when clinicoradiological findings are suggestive of PML, even with initially normal CSF studies.

## 2. Case

**Figure 1:**
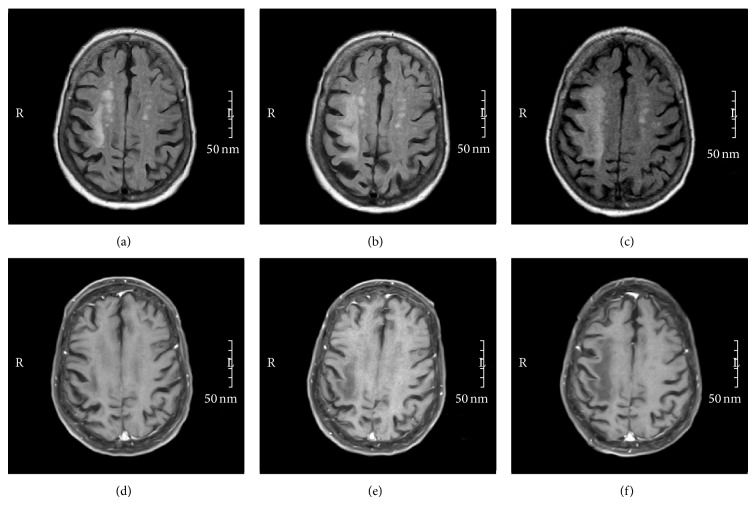
Top row: MRI head, axial T2-FLAIR. Bottom row: MRI head, T1-post gadolinium contrast in same chronological order (right to left). Note the prominent progressive asymmetric subcortical T2-FLAIR signal abnormality over the course of 12 weeks with noted lack of pathological enhancement during the same time window. (Left) image: week 1, middle: week 6, and right: week 12. Lumbar puncture was obtained after the second MRI (middle).

A 75-year-old woman with history of rheumatoid arthritis maintained on long term methotrexate and adalimumab presented with a progressive left hemiplegia and global decline. Initial magnetic resonance imaging (MRI) of the brain without and with contrast (Figures 1(a) and 1(d)) demonstrated a right subcortical T2-FLAIR hyperintense lesion with negative diffusion-restriction correlate and was attributed to a possible age indeterminate infarction. Less severe left hemispheric periventricular white matter involvement was also identified and thought to be reflective of chronic ischemic white matter disease. Because of the worsening of neurological examination as well as radiologic progression of right hemispheric lesion (Figures 1(b) and 1(e)), a lumbar puncture (LP) obtained 1 month after her initial presentation was normal including PCR for JC virus.

**Figure 2:**
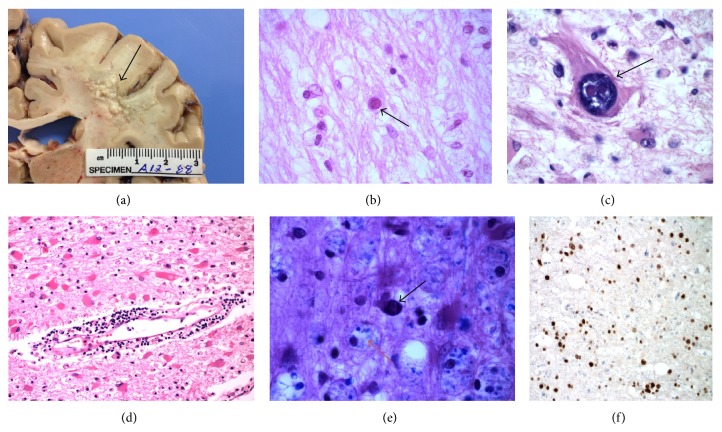
(a) PML gross pathology finding, right frontoparietal white matter lesion (black arrow). The subcortical whiter matter shows an irregular disruption measuring 1 × 1.5 cm. (b) Viral inclusions in an enlarged oligodendroglial nucleus (black arrow). (c) Bizarre reactive astrocyte with enlarged nucleus and prominent nucleolus. (d) Perivascular cuffing by lymphocytes. (e) Luxol Fast Blue/H&E stain showing an intranuclear inclusion (black arrow) and numerous foamy macrophages containing myelin debris (red arrow). (f) Immunocytochemistry using target-antibody against SV40, showing numerous positively stained oligodendroglia.

The patient continued to decline rapidly with confirmation on serial MRI (Figures 1(c) and 1(f)). She expired one day prior to a planned repeat lumbar puncture, approximately 3 months from the onset of her symptoms. Autopsy confirmed PML and the presence of JC virus by immunohistochemical staining (Figure 2).

## 3. Discussion

Our patient's MRI showed typical findings of PML, including bilateral asymmetric nonenhancing multifocal areas of periventricular and subcortical white matter demyelination that did not conform to the typical cerebral arterial distributions, along with lack of mass effect and U-fibers involvement. Unlike many prior reported cases, where, in the setting of subtle MRI findings, JC virus CSF PCR was diagnostic [1, 2, 9, 10], our case had a markedly abnormal MRI (Figures 1(c) and 1(f)) and clinical findings suggestive of progressive disease, yet with negative CSF studies, thus highlighting the potential of false-negative CSF JC virus PCR.

Plausible explanations for the false-negative JC virus analysis of CSF studies include inadequate choice of JC virus DNA PCR targets [13], low viral DNA in the CSF, or utilization of different detection thresholds [8]. Ultrasensitive TaqMan real-time PCR assay (available at National Institutes of Health and other international laboratories) detects fewer viral loads >10 DNA copies/mL, whereas other readily available quantitative real-time PCR techniques such as those used in this case have a reported threshold of >50 DNA copies/mL. Other possible explanations for the false-negative result include low volume of the specimen, loss of DNA during concentration, or lack of spontaneous dissemination into the subarachnoid space [3, 9].

We suggest that when the clinical presentation is suspicious of PML, a negative initial CSF JC virus PCR result does not exclude such diagnosis. Therefore, consideration for repeat CSF analysis, utilizing ultrasensitive PCR assay, should be undertaken, and exclusion of other potential masquerading neurologic conditions is essential. These conditions include autoimmune and inflammatory conditions such as multiple sclerosis and acute disseminated encephalomyelitis (ADEM), infectious conditions such as HIV encephalitis and Varicella-Zoster virus encephalopathy, and other conditions such as mitochondrial encephalopathies, CNS vasculitis, and reversible posterior leukoencephalopathy [14, 15]. If, however, ascertaining the diagnosis of PML remains elusive, then a brain biopsy should be undertaken. This remains the gold standard for the diagnosis of PML, specifically when clinical and neuroimaging features are suggestive of PML despite negative JC virus CSF PCR analysis.
